# Long-term survival and clinical characteristics of colon and rectal cancer: a comprehensive 47-year retrospective analysis using the SEER database

**DOI:** 10.1097/MS9.0000000000005179

**Published:** 2026-06-02

**Authors:** Mohammad Al Diab Al Azzawi, Abdulrahman S. Binsaleh, Munira Hatem Alsharif, Meshal Fahad Aldhilan, Sara Hamed Abusabaah, Marya Ayman Alfurayj, Eyesha Aadham Junaidallah, Lajeen Naser Alnowaisser, Sarah Abdulaziz Alsaggaf

**Affiliations:** a Faculty of Medicine, The National Ribat University, Khartoum, Sudan; b College of Medicine, Prince Sattam Bin Abdulaziz University, Al-Kharj, Saudi Arabia; c College of Medicine, King Saud University, Riyadh, Saudi Arabia; d College of Medicine, Qassim University, Qassim, Saudi Arabia; e College of Medicine, Almaarefa University, Riyadh, Saudi Arabia; f School of Medicine, University College Dublin, Dublin, Ireland; g Faculty of Pharmacy, King Abdulaziz University, Jeddah, Saudi Arabia

**Keywords:** cancer epidemiology, colorectal cancer, long-term survival, population-based study, SEER database

## Abstract

**Background:**

Population screening and therapeutic advances have changed colorectal cancer (CRC) incidence and survival over recent decades, yet important differences exist by anatomic subsite, histology, and population subgroup. This study describes long-term trends in incidence and survival for colon and rectal tumors.

**Methods:**

We analyzed 501 094 primary colon and rectal tumor records from the Surveillance, Epidemiology, and End Results (SEER) Incidence database (1975–2022). Age-adjusted rates used the 2000 U.S. standard population. Outcomes were overall survival (OS) and CRC-specific mortality. OS was estimated using Kaplan–Meier methods and compared between groups by log-rank tests. CRC-specific mortality was evaluated using cause-specific Cox regression based on SEER cause-of-death recodes, adjusting for age, sex, race, histology, primary site, and surgery status.

**Results:**

Age-adjusted incidence was 48.3 per 100 000. Median OS improved across eras [Era 1 (1975–1994) 3.83 years, Era 2 (1995–2009) 5.58 years, and Era 3 (2010–2022) 6.92 years, all Eras had *P* value < 0.001). Left- versus right-sided colon cancers and rectal versus colon primaries showed fixed survival differences across eras. Regarding histology, the signet-ring tumors had markedly worse survival, while neuroendocrine/carcinoid tumors showed better long-term survival. In the adjusted Cox model, several colonic subsites showed a higher hazard of CRC-specific death compared to the appendix. Signet-ring cell histology conferred the greatest mortality risk (HR = 2.206, 95% CI 2.113–2.304; *P* < 0.001).

**Conclusions:**

Over a 47-year period, survival in CRC has significantly improved, but outcomes remain influenced by anatomic subsite and histology. These findings confirm that tumor subsite and histology remain important determinants of long-term prognosis in CRC and highlight the need for risk-adapted management strategies, continued population-based surveillance, and ongoing efforts to address disparities in outcomes.

## Introduction

Colorectal cancer (CRC) is one of the leading causes of morbidity and mortality. Globally, it accounts for about two million incident cases and nearly one million deaths each year. This places CRC among the most diagnosed cancers worldwide^[^1^]^. Recent national registry summaries report an age-adjusted incidence rate of 37 per 100 000. The current 5-year relative survival rate is near two-thirds overall^[^2^]^.

Despite improvements in screening, earlier detection, and multidisciplinary care, which have been associated with a declining incidence of almost 1% every year among older adults, the rate of early-onset CRC in individuals under 50 has been rising by 1–2% each year since the early 1990s. This represents nearly 20 000 cases of CRC in young patients and 4000 deaths annually within that demographic^[^3,4^]^.

Significant heterogeneity in CRC outcomes persists across stage at diagnosis, anatomic site, and population subgroups. Stage remains the dominant prognostic determinant. Contemporary U.S. estimates show 5-year relative survival of about 91% for localized disease versus 14% for distant disease^[^4^]^. The Surveillance, Epidemiology, and End Results (SEER)-based analyses indicate persistently lower stage-specific survival probabilities for American Indians, Alaska Natives, and non-Hispanic Black patients across multiple stages. These patterns highlight enduring inequities, even as overall survival (OS) improves^[^5^]^. Studies demonstrate distinctions between rectal and colon cancer and, within colon cancer, between right- and left-sided primaries. Left-sided colon cancers have better survival in the era of modern therapy^[^6^]^. Therapeutic developments since the 1970s, such as surgical refinements, improved perioperative care, and enhanced systemic regimens, have been associated with improved survival^[^7^]^. In particular, the development of immunotherapy for the subgroup of tumors with deficient mismatch repair/high microsatellite instability (dMMR/MSI-H) has transformed standards for selectively identified patients. The phase III study of KEYNOTE-177 demonstrated durable efficacy for the first-line use of pembrolizumab in MSI-H/dMMR metastatic CRC, with durable responses and favorable toxicity profile on long-term follow-up relative to chemotherapy^[^8,9^]^. Over the same period, survival gains have coincided with refinements in colorectal and rectal cancer surgery, perioperative and supportive care, and successive generations of systemic therapy, including combination chemotherapy, biologic agents, and immunotherapy in selected molecular subgroups^[^7^]^.

Long-term population-based evaluations are essential to determine how incidence, stage distribution, survival, and disparities have shifted with screening policy and therapeutic innovation. The SEER program provides a comprehensive and standardized dataset spanning multiple decades. Building on prior SEER investigations, this 47-year retrospective analysis (1975–2022) will: (1) describe age-adjusted incidence and case distribution across calendar periods for colon and rectal cancers; (2) characterize long-term survival patterns by anatomic subsite (colon vs rectum; left- vs right-sided colon) and histologic subtype; and (3) explore racial/ethnic differences in CRC-specific mortality using multivariable models, while recognizing that detailed stage-specific analyses are beyond the scope of this report.

## Methods

### Protocol registration and guideline criteria

The study protocol was registered with the Open Science Framework. Although this is a retrospective analysis of the SEER database, the registration was conducted a priori to ensure methodological transparency and reproducibility. The reporting of this work was aligned with the STROCSS criteria and fulfilled the requirements of the TITAN Guidelines 2025^[^10,11^]^.

### Data source

We used population-based cancer registry data from the SEER Program (seer.cancer.gov). Analyses were performed in SEER*Stat v9.0.41 using the Incidence – SEER Research Data, 8 Registries, November 2024 Submission (1975–2022), released in April 2025. Rates were age-adjusted to the 2000 U.S. standard population^[^12^]^. We restricted the analysis to the SEER 8 registries to maintain consistent geographic coverage across the entire 1975–2022 period and avoid artifacts from changes in registry catchment. Although larger SEER datasets (SEER 9, 13, or 18) provide broader population coverage, their phased expansion over time would have introduced discontinuities in geographic representation, potentially confounding long-term temporal trend analyses. We therefore prioritized temporal consistency across the full 47-year study period, acknowledging the trade-off of reduced national coverage. SEER 8 covers approximately 8% of the U.S. population, and although these registries were selected to be broadly representative and to oversample certain racial and ethnic groups, the results may not fully generalize to non-SEER regions.

HIGHLIGHTS

- This 47-year retrospective cohort analysis of 501 094 colorectal cancer (CRC) cases from the Surveillance, Epidemiology, and End Results database (1975–2022) provides a large long-term population-based evaluation.
- Median overall survival in CRC improved substantially across eras—from 3.8 years (1975–1994) to 6.9 years (2010–2022), reflecting advances in screening and therapy.
- Left-sided colon cancers consistently showed better survival than right-sided primaries, while rectal cancers demonstrated improved outcomes relative to colon cancers in recent decades.
- Histologic subtype strongly predicted survival: signet-ring carcinoma conferred the highest mortality risk, whereas neuroendocrine/carcinoid tumors had the most favorable outcomes.
- Findings highlight the enduring prognostic value of tumor site, histology, and surgery, supporting ongoing tailored surveillance and treatment strategies in CRC.

### Study population

All cases with a primary tumor of the colon or rectum were identified using the SEER variables Site recode ICD-O-3/WHO 2008 and Primary Site–labeled, and all codes for the colon and rectum available are: C18.0-Cecum, C18.1-Appendix, C18.2-Ascending colon, C18.3-Hepatic flexure of colon, C18.4-Transverse colon, C18.5-Splenic flexure of colon, C18.6-Descending colon, C18.7-Sigmoid colon, C18.8-Overlapping lesion of colon, C18.9-Colon, not otherwise specified (NOS), C19.9-Rectosigmoid junction, C20.9-Rectum, NOS, and C26.0-Intestinal tract, NOS. Patients were excluded if information on survival time, vital status, or diagnostic confirmation was missing. In the extracted cohort (*N* = 501 094), no cases were excluded for missing survival time, vital status, or diagnostic confirmation (0%).

### Variables collected

The following SEER variables were incorporated:

1. Demographic variables: Age recode with <1-year-olds and 90+ years; Age recode with single ages and 85+ years; Sex; Race recode (White, Black, Other, Unknown); and Year of diagnosis. The SEER race recode variable aggregates American Indian/Alaska Native and Asian/Pacific Islander patients into a single “Other” category to ensure consistent coding across years.
2. Tumor characteristics: Site recode ICD-O-3/WHO 2008, Primary Site–labeled, ICD-O-3 Histology/Behavior, and Diagnostic Confirmation. Right-sided colon cancer was defined as cecum (C18.0), ascending colon (C18.2), hepatic flexure (C18.3), and transverse colon (C18.4). Left-sided colon cancer was defined as splenic flexure (C18.5), descending colon (C18.6), sigmoid colon (C18.7), and rectosigmoid junction (C19.9), with splenic flexure categorized as left-sided. Appendix (C18.1) and non-specific/overlapping categories (C18.8, C18.9, C26.0) were not assigned laterality and were excluded from right–left comparisons; rectal cancers (C20.9) were analyzed separately.
3. Tumor stage (sensitivity analyses): Tumor stage was obtained from the SEER variable Combined Summary Stage with Expanded Regional Codes (2004+) and collapsed into three categories: localized, regional, and distant disease.
4. Treatment-related variables: Reason no cancer-directed surgery (to capture surgical treatment status).
5. Survival outcomes: Survival months, vital status recodes (study cutoff used), and COD-to-site recode.
6. Follow-up information: Year of follow-up recode.

### Outcomes

The primary outcome was OS, defined as the time from diagnosis to death from any cause or the last follow-up. CRC-specific mortality (CSS) was a secondary outcome, evaluated using cause-specific Cox regression based on SEER cause-of-death recodes for colon and rectal tumors.

### Statistical analysis

Baseline characteristics were described using summary statistics. Age-adjusted incidence rates (per 100 000) were calculated in SEER*Stat using direct standardization to the 2000 U.S. standard population (age <1 and 90 +). Incidence was summarized overall and stratified by diagnostic era (1975–1994, 1995–2009, 2010–2022), age group (<50 vs ≥50 years), sex, race, and anatomic site. Survival analyses were performed using the Kaplan–Meier method, with survival differences compared using the log-rank test. Independent prognostic factors were assessed with Cox proportional hazards regression models, adjusting for age (continuous, per 1-year increase), sex, race, histology, primary site, and surgery status. In sensitivity analyses, stage and diagnostic era were additionally evaluated to account for temporal and disease-related confounding. We report median OS and survival probabilities at fixed time points; because follow-up is substantially shorter and right-censoring heavier for patients diagnosed in 2010–2022, mean OS for this era is not reported or used for interpretation. Long-term survival trends were evaluated across decades of diagnosis. Diagnostic eras were defined a priori to reflect broad phases in CRC screening and treatment evolution while maintaining adequate follow-up within each period. The 1975–1994 era represents the pre-widespread colonoscopy screening period and early chemotherapy era; 1995–2009 corresponds to increasing uptake of colonoscopy and the adoption of modern adjuvant and combination systemic therapies; and 2010–2022 reflects the contemporary era characterized by biologic agents, immunotherapy for selected molecular subgroups, and further refinements in multidisciplinary care. Although the eras are not equal in duration, they were selected to balance clinical relevance and sufficient case numbers rather than to imply discrete biologic transitions. Analyses were conducted using SEER*Stat 9.0.41 and SPSS software version 25, with statistical significance set at two-sided *P* < 0.05. In addition, a sensitivity cause-specific Cox regression restricted to patients <50 years was performed to evaluate CRC-specific mortality in early-onset disease.

## Result

### Epidemiology

**Figure 1.**
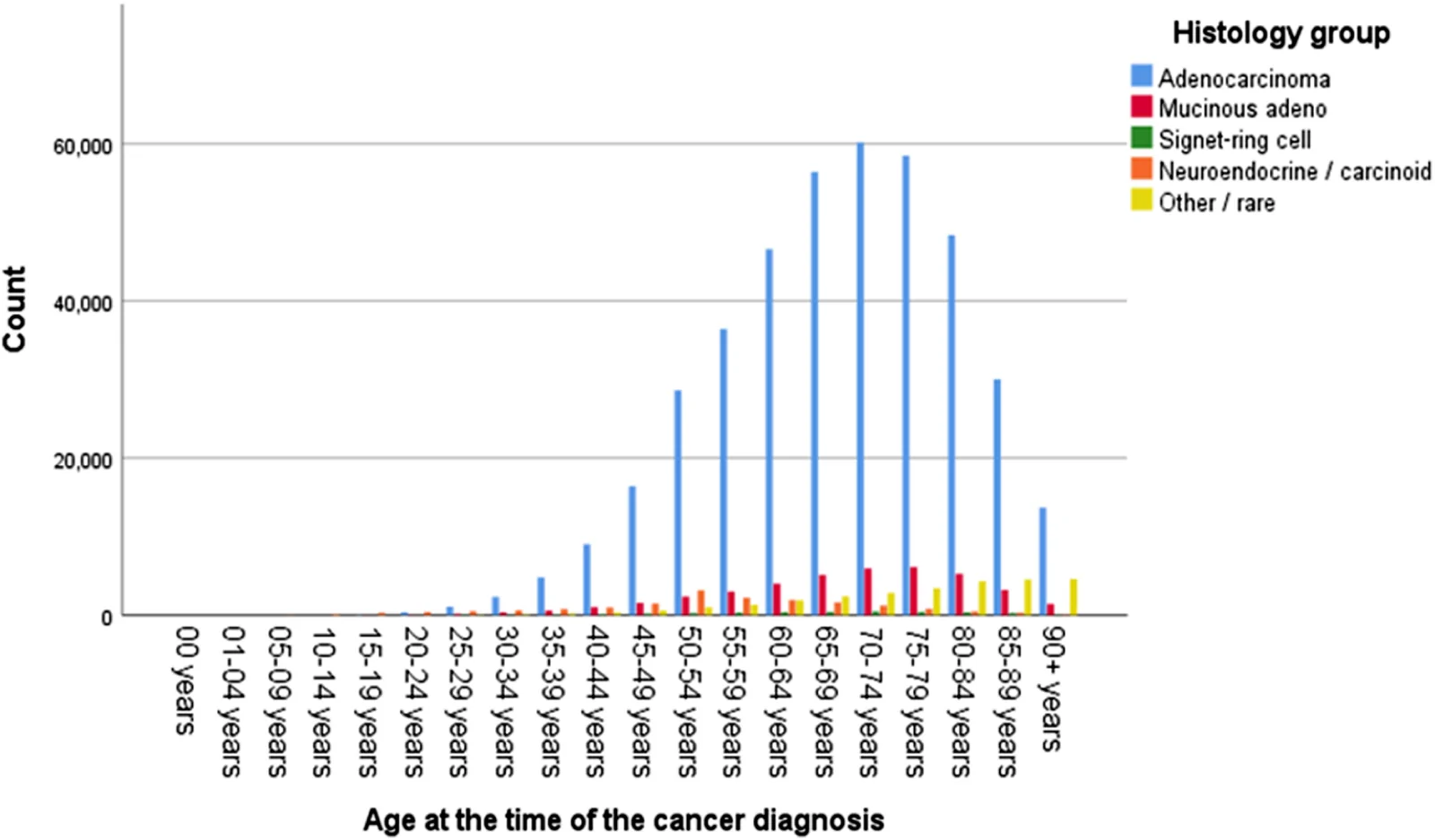
Age distribution of colorectal cancer at diagnosis by histologic subtype.

Among 501 094 patients, most were diagnosed at older ages: 24.1% at 60–69 years and 27.9% at 70–79 years (52.0% combined), with 19.4% at 80–89, 15.7% at 50–59, 6.3% at 40–49, 2.6% under 40, and 4.0% ≥ 90; the age distribution is shown in Figure 1. Sex distribution was balanced (male 50.8%, female 49.2%). By race, 83.1% were White, 7.0% Black, 9.5% were categorized as Other (including American Indian/Alaska Native and Asian/Pacific Islander), and 0.4% were Unknown (Table 1). Anatomically, 79.3% were colon (C18.x/C19.9) and 20.7% rectum (C20.9). Among colon tumors with assignable laterality, right- and left-sided primaries were nearly equal (50.3% vs 49.7%). Histologically, adenocarcinoma predominated (82.4%), followed by mucinous adenocarcinoma (8.0%), neuroendocrine/carcinoid (3.4%), signet-ring cell (0.7%), and other/rare types (5.5%); complete histopathology groupings and codes are provided in Supplemental Digital Content Table S1, available at: http://links.lww.com/MS9/B226. Cases were distributed across calendar periods as 9.4% (1975–1984), 15.0% (1985–1994), 18.3% (1995–2004), 19.0% (2005–2014), and 38.2% (2015–2022). Diagnostic confirmation was histologic in 96.1%, with 2.7% other/non-histologic methods and 1.2% unknown. Overall, the age-adjusted incidence was 48.3 per 100 000 (95% CI 48.1–48.4), and incidence was higher in males than females (55.6 [95% CI 55.3–55.8] vs 42.5 [95% CI 42.3–42.6] per 100 000). By race, age-adjusted incidence rates were 48.6 (95% CI 48.5–48.8) in White patients, 51.7 (95% CI 51.1–52.3) in Black patients, and 40.6 (95% CI 40.2–40.9) in the Other race category. Across diagnostic eras, overall age-adjusted incidence declined from 60.9 per 100 000 in Era 1 (1975–1994) to 50.2 in Era 2 (1995–2009) and 36.6 in Era 3 (2010–2022); however, trends differed by age group. Among individuals <50 years, incidence increased from 5.3 per 100 000 in Era 1 to 5.9 in Era 2 and 8.2 in Era 3, whereas among individuals ≥50 years, incidence declined from 206.6 to 166.2 to 110.9 per 100 000. Incidence declined across eras in both males (72.5 to 58.5 to 41.3 per 100 000) and females (52.9 to 43.6 to 32.5 per 100 000) and decreased over time within major racial groups (White 61.5 to 50.0 to 36.0; Black 61.9 to 57.8 to 42.5; Other 50.3 to 44.9 to 34.1 per 100 000). By anatomic site, incidence declined for colon excluding rectum (43.9 to 36.4 to 25.5 per 100 000) and for rectum/rectosigmoid junction (17.1 to 13.8 to 11.1 per 100 000), while appendiceal cancers increased (0.4 to 0.6 to 1.6 per 100 000) (Supplemental Digital Content Table S2, available at: http://links.lww.com/MS9/B226).

**Table 1:** The characteristics of the 501 094 study patients.

Characteristic	Category	*n*	%^a^
Age at diagnosis^b^	< 40 y	13 195	2.6
	40–49 y	31 694	6.3
	50–59 y	78 691	15.7
	60–69 y	120 783	24.1
	70–79 y	139 820	27.9
	80–89 y	97 056	19.4
	≥ 90 y	19 855	4.0
Sex	Female	246 518	49.2
	Male	254 576	50.8
Race	White	416 523	83.1
	Black	35 159	7.0
	Other (AI/AN & API)	47 549	9.5
	Unknown	1863	0.4
Year of diagnosis period	1975–1984	47 111	9.4
	1985–1994	75 263	15.0
	1995–2004	91 781	18.3
	2005–2014	95 333	19.0
	2015–2022	191 606	38.2
Diagnostic confirmation	Positive histology	481 646	96.1
	Other/non-histologic^c^	13 548	2.7
	Unknown	5900	1.2

^a^ Percentages are calculated out of the total cohort (*N* = 501 094).

^b^ Derived from SEER “Age recode <1 y & 90+” field; categories collapsed for readability.

^c^ Includes clinical diagnosis only, direct visualization without histology, positive cytology, radiography only, laboratory marker, and unspecified microscopic confirmation.

### OS across eras

Across the cohort, OS differed significantly by era (log-rank *P* < 0.001). Median OS was 3.83 years (95% CI 3.79–3.88) in Era 1 (1975–1994), 5.58 years (95% CI 5.52–5.65) in Era 2 (1995–2009), and 6.92 years (95% CI 6.81–7.02) in Era 3 (2010–2022).

### OS for colon vs rectum

**Figure 2.**
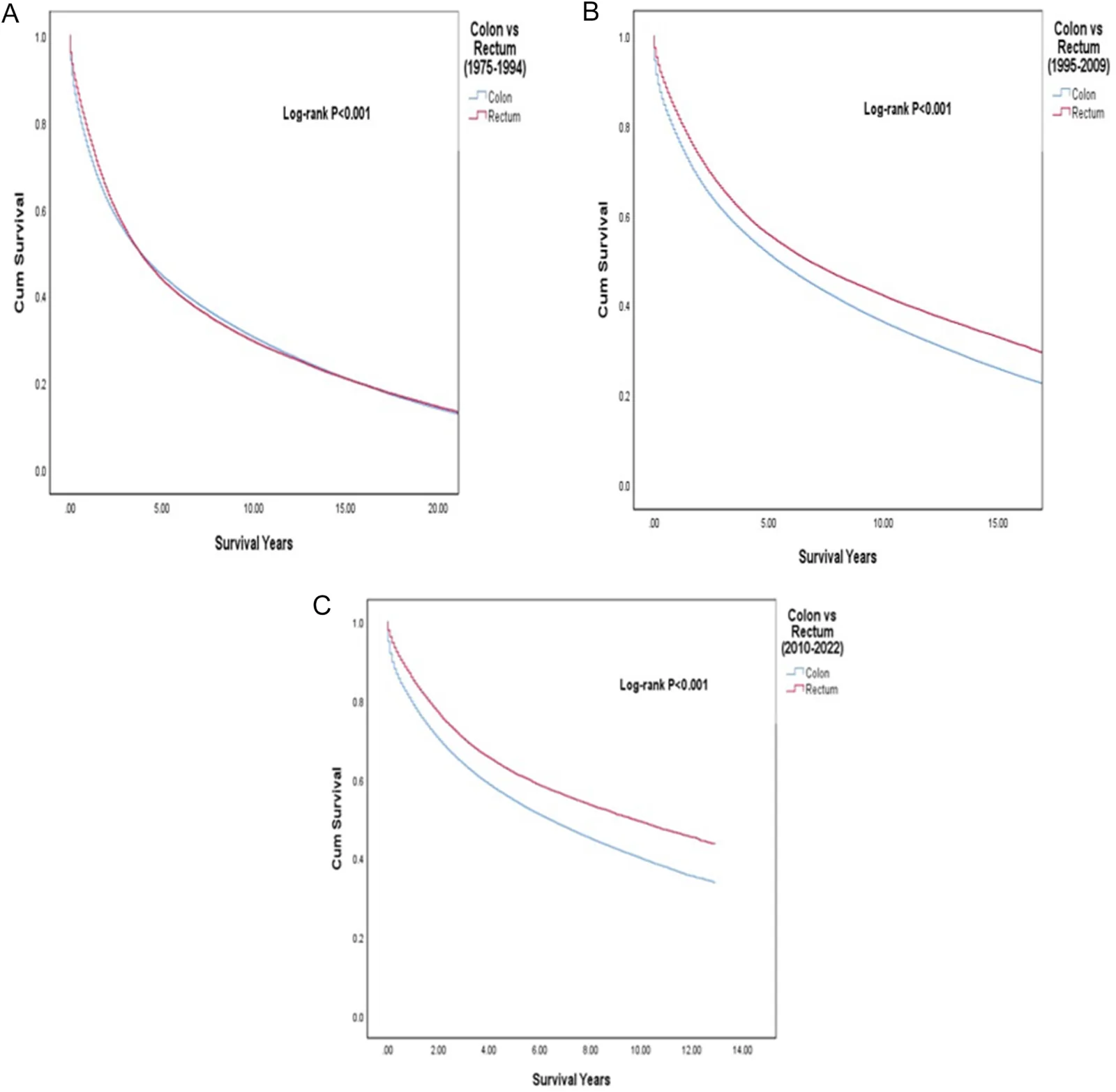
Overall survival by colon vs rectum for period A (1975–1994) (A), period B (1995–2009) (B), and period C (2010–2022) (C).

Era 1 (1975–1994): Median survival was 3.83 years for colon cancer (95% CI 3.78–3.88) and 3.83 years for rectal cancer (95% CI 3.75–3.92); the 5-year OS was 45% and 44%, respectively. Era 2 (1995–2009): Median survival was 5.33 years for colon cancer (95% CI 5.26–5.40) and 6.67 years for rectal cancer (95% CI 6.48–6.85); the 5-year OS was 52% and 56%, respectively. Era 3 (2010–2022): Median survival was 6.33 years for colon cancer (95% CI 6.23–6.44) and 9.58 years for rectal cancer (95% CI 9.24–9.93); the 5-year OS was 55% and 62%, respectively. Log-rank tests comparing colon versus rectal cancer survival were significant within every era (Era 1: *P* < 0.001; Era 2: *P* < 0.001; Era 3: *P* < 0.001) (Fig. 2).

### OS for left- vs right-side colon

**Figure 3.**
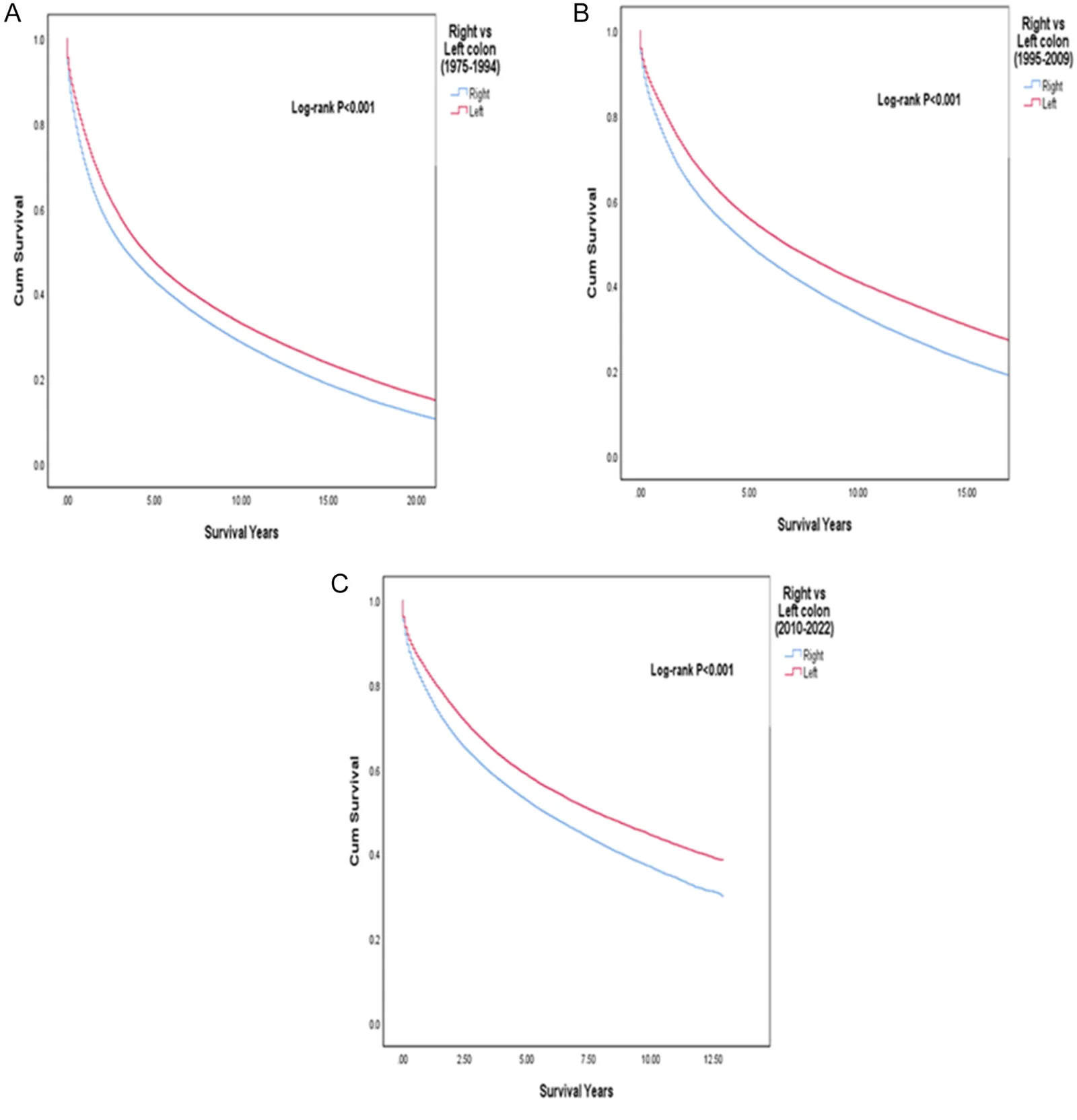
Overall survival by left vs right colon for period A (1975–1994) (A), period B (1995–2009) (B), and period C (2010–2022) (C).

Era 1 (1975–1994): Median survival was 3.42 years for right-sided colon (95% CI 3.34–3.49) and 4.42 years for left-sided colon (95% CI 4.34–4.49); the 5-year OS was 43% and 48%, respectively. Era 2 (1995–2009): Median survival was 4.92 years for right-sided colon (95% CI 4.83–5.01) and 6.58 years for left-sided colon (95% CI 6.46–6.71); the 5-year OS was 50% and 56%, respectively. Era 3 (2010–2022): Median survival was 5.67 years for right-sided colon (95% CI 5.53–5.80) and 7.75 years for left-sided colon (95% CI 7.53–7.97); the 5-year OS was 52% and 59%, respectively. Log-rank tests comparing left- versus right-sided colon cancer survival were significant within every era (Era 1: *P* < 0.001; Era 2: *P* < 0.001; Era 3: *P* < 0.001) (Fig. 3).

### The OS of histology types

**Figure 4.**
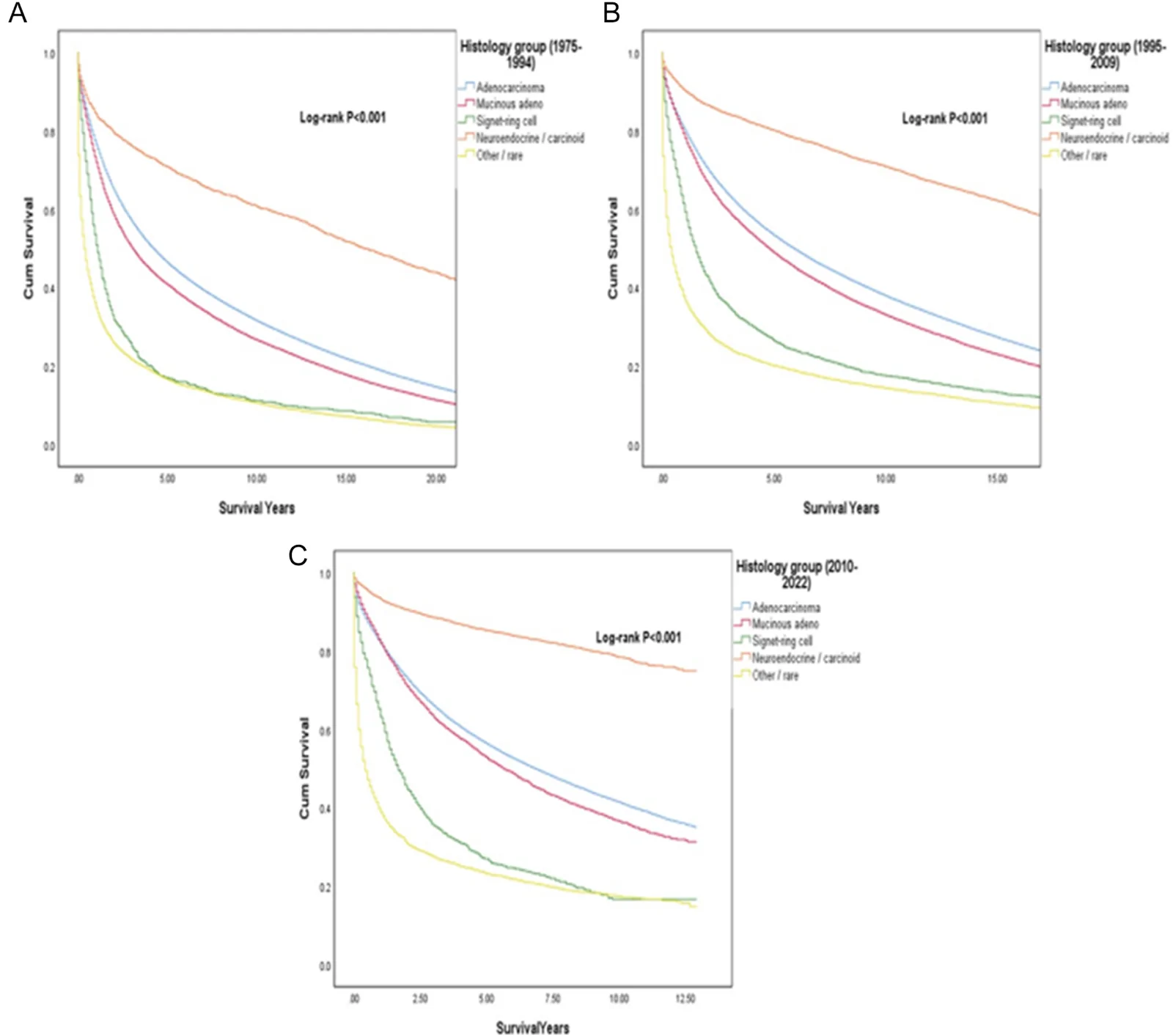
Overall survival by histology type for period A (1975–1994) (A), period B (1995–2009) (B), and period C (2010–2022) (C).

Different histology types were grouped into five main categories (Supplemental Digital Content Table S1, available at: http://links.lww.com/MS9/B226). By histologic subtype, survival differed within each era (log-rank *P* < 0.001 across subtypes). In Era 1 (1975–1994), the median OS was: adenocarcinoma 4.25 years, mucinous adenocarcinoma 3.08 years, signet-ring cell 1.08 years, neuroendocrine/carcinoid 16.00 years, and other/rare 0.33 years; the corresponding 5-year OS was 47, 41, 17, 71, and 17%, respectively. In Era 2 (1995–2009), the median OS was: adenocarcinoma 5.92 years, mucinous 4.75 years, signet-ring 1.50 years, neuroendocrine/carcinoid 21.75 years, and other/rare 0.42 years; the 5-year OS was 54, 49, 27, 80, and 20%, respectively. In Era 3 (2010–2022), the median OS was: adenocarcinoma 6.83 years, mucinous adenocarcinoma 5.67 years, signet-ring 1.67 years, neuroendocrine/carcinoid not reached, and other/rare 0.42 years; the 5-year OS was 57, 54, 28, 85, and 22%, respectively (Fig. 4).

### The OS of reason for no cancer-directed surgery

**Figure 5.**
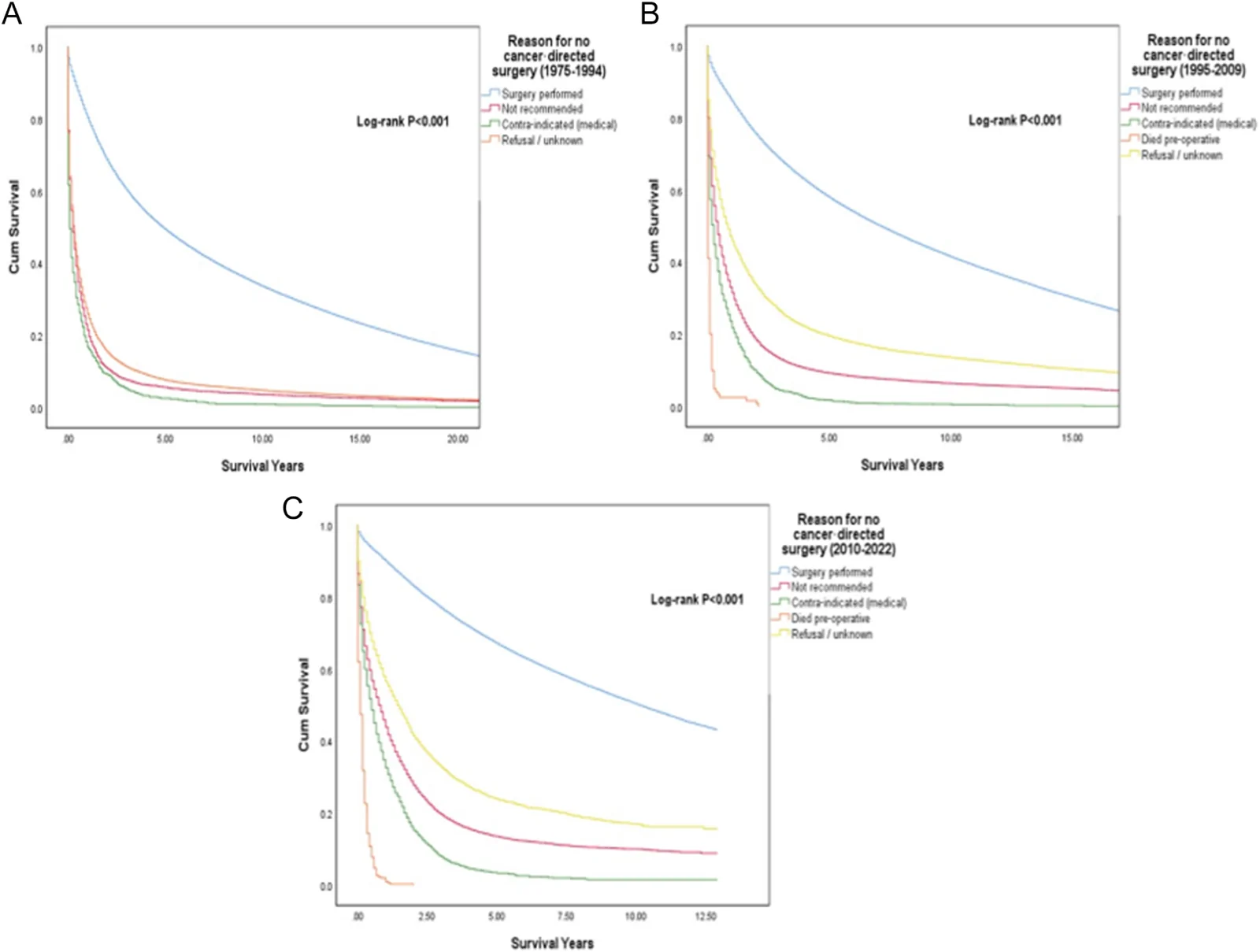
Overall survival by reason for no cancer-directed surgery during period A (1975–1994) (A), period B (1995–2009) (B), and period C (2010–2022) (C).

Survival differed significantly by surgery category within each era (log-rank *P* < 0.001 across categories). In Era 1 (1975–1994), median OS by surgery category was: surgery performed, 4.92 years; not recommended, 0.25 years; contraindicated, 0.08 years; and refusal/unknown, 0.33 years. The corresponding 5-year OS was 50, 6, 3, and 8%, respectively. In Era 2 (1995–2009), median OS was: surgery performed, 7.17 years; not recommended, 0.42 years; contraindicated, 0.25 years; and refusal/unknown, 0.83 years. The 5-year OS was 58, 10, 2, and 20%, respectively. In Era 3 (2010–2022), median OS was: surgery performed, 10.17 years; not recommended, 0.83 years; contraindicated, 0.50 years; and refusal/unknown, 1.42 years. The 5-year OS was 68, 17, 7, and 26, respectively. Five-year OS could not be estimated for the died pre-operative group due to the absence of valid survival time (Fig. 5).

### The OS of primary site

**Figure 6.**
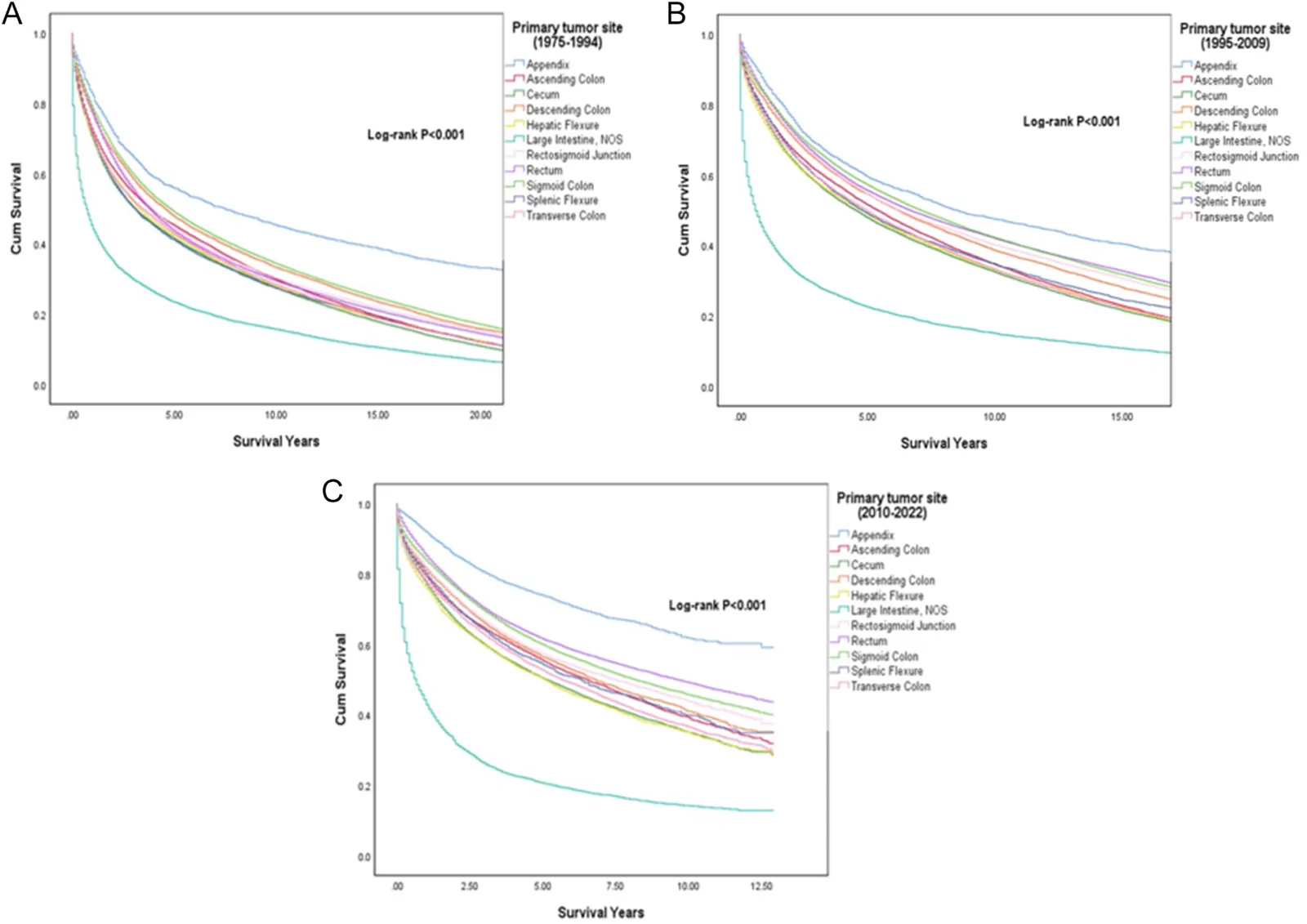
Overall survival by primary tumor site for period A (1975–1994) (A), period B (1995–2009) (B), and period C (2010–2022) (C).

By primary anatomic site, survival differed within each era (log-rank *P* < 0.001 across sites). In Era 1 (1975–1994), median OS (years) and 5-year OS were as follows: appendix, 7.50 years and 56%; ascending colon, 3.83 years and 45%; cecum, 3.08 years and 42%; descending colon, 4.58 years and 48%; hepatic flexure, 3.33 years and 43%; large intestine NOS, 0.75 years and 24%; rectosigmoid junction, 3.92 years and 45%; rectum, 3.83 years and 44%; sigmoid colon, 4.83 years and 49%; splenic flexure, 3.08 years and 41%; and transverse colon, 3.42 years and 43%. In Era 2 (1995–2009), median OS and 5-year OS were as follows: appendix, 8.50 years and 60%; ascending colon, 5.42 years and 52%; cecum, 4.58 years and 48%; descending colon, 6.17 years and 55%; hepatic flexure, 4.75 years and 49%; large intestine NOS, 0.58 years and 23%; rectosigmoid junction, 6.25 years and 55%; rectum, 6.67 years and 56%; sigmoid colon, 7.08 years and 58%; splenic flexure, 4.83 years and 49%; and transverse colon, 5.00 years and 50%. In Era 3 (2010–2022), median OS and 5-year OS were as follows: appendix, median not reached with 5-year OS of 74%; ascending colon, 6.50 years and 55%; cecum, 5.17 years and 50%; descending colon, 6.83 years and 57%; hepatic flexure, 5.08 years and 50%; large intestine NOS, 0.67 years and 21%; rectosigmoid junction, 7.50 years and 57%; rectum, 9.58 years and 62%; sigmoid colon, 8.17 years and 60%; splenic flexure, 6.33 years and 54%; and transverse colon, 5.67 years and 53% (Fig. 6).

### The overall stage-stratified survival

In the subset of 195 324 tumors with available combined summary stage (localized, regional, distant), OS differed markedly by stage (log-rank *P* < 0.001). Median OS was 12.67 years for localized disease (95% CI 12.51–12.83), 8.00 years for regional disease (95% CI 7.87–8.13), and 1.08 years for distant disease (95% CI 1.06–1.11). The corresponding 5-year OS was 76% for localized disease, 62% for regional disease, and 16% for distant disease. Supplemental Digital Content Figure S7, available at: http://links.lww.com/MS9/B226

### Multivariable Cox model for cause-specific CRC mortality

In the multivariable cause-specific Cox model for CRC mortality (event = CRC death; other causes censored), the overall model was significant (overall *P* < 0.001), and each covariate set was significant overall. Female sex was associated with a lower hazard of CRC-specific death compared with males [hazard ratio (HR) = 0.955, 95% confidence interval (CI) 0.946–0.964; *P* < 0.001]. Using White patients as the reference, Black patients had a higher hazard (HR = 1.101, 95% CI 1.081–1.121; *P* < 0.001), whereas the “Other” race category (American Indian/Alaska Native or Asian/Pacific Islander) had a lower hazard (HR = 0.888, 95% CI 0.873–0.903; *P* < 0.001); the unknown race category also showed a lower hazard (HR = 0.234, 95% CI 0.200–0.275; *P* < 0.001). Relative to adenocarcinoma, mucinous adenocarcinoma (HR = 1.277, 95% CI 1.255–1.298; *P* < 0.001), signet-ring cell carcinoma (HR = 2.206, 95% CI 2.113–2.304; *P* < 0.001), and other/rare histologies (HR = 1.221, 95% CI 1.196–1.246; *P* < 0.001) were associated with higher CRC-specific mortality, while neuroendocrine/carcinoid tumors were associated with lower mortality (HR = 0.211, 95% CI 0.200–0.223; *P* < 0.001). Compared with patients who underwent cancer-directed surgery, CRC-specific mortality was higher when surgery was not recommended (HR = 5.631, 95% CI 5.547–5.716; *P* < 0.001), contraindicated for medical reasons (HR = 6.229, 95% CI 6.002–6.466; *P* < 0.001), not performed due to pre-operative death (HR = 12.822, 95% CI 11.268–14.590; *P* < 0.001), or refusal/unknown (HR = 4.609, 95% CI 4.532–4.688; *P* < 0.001). Using appendix as the reference, hazards were higher for cecum (HR = 1.160, *P* < 0.001), large intestine NOS (HR = 1.289, *P* < 0.001), rectosigmoid junction (HR = 1.143, *P* < 0.001), splenic flexure (HR = 1.238, *P* < 0.001), and transverse colon (HR = 1.087, *P* = 0.001), lower for ascending colon (HR = 0.946, *P* = 0.027) and rectum (HR = 0.906, *P* < 0.001), and not significantly different for descending colon (*P* = 0.067), hepatic flexure (*P* = 0.059), or sigmoid colon (*P* = 0.858). Age at diagnosis was independently associated with CRC-specific mortality, with a 1.3% increase in hazard per additional year (HR = 1.013, 95% CI 1.013–1.014; *P* < 0.001) (Table 2). Because the unknown race category showed an implausible hazard ratio, we repeated the model after excluding Unknown race; results were materially unchanged (Supplemental Digital Content Table S3, available at: http://links.lww.com/MS9/B226).

**Table 2:** Multivariable Cox model for cause-specific colorectal cancer mortality.

Variable	Category	Hazard ratio (HR)	95% CI	*P*-value
Age	Per 1-year increase	1.013	1.013–1.014	<0.001
Sex	Male	1.00 (reference)	–	–
	Female	0.955	0.946–0.964	<0.001
Race	White	1.00 (reference)	–	–
	Black	1.101	1.081–1.121	<0.001
	Other (AI/AN, API)	0.888	0.873–0.903	<0.001
	Unknown	0.234	0.200–0.275	<0.001
Histology	Adenocarcinoma	1.00 (reference)	–	–
	Mucinous adenocarcinoma	1.277	1.255–1.298	<0.001
	Signet-ring cell	2.206	2.113–2.304	<0.001
	Neuroendocrine/carcinoid	0.211	0.200–0.223	<0.001
	Other/rare	1.221	1.196–1.246	<0.001
Reason no cancer-directed surgery	Surgery performed	1.00 (reference)	–	–
	Not recommended	5.631	5.547–5.716	<0.001
	Contra-indicated (medical)	6.229	6.002–6.466	<0.001
	Died pre-operative	12.822	11.268–14.590	<0.001
	Refusal/unknown	4.609	4.532–4.688	<0.001
Primary site	Appendix	1.00 (reference)	–	–
	Ascending colon	0.946	0.901–0.994	0.027
	Cecum	1.160	1.106–1.217	<0.001
	Descending colon	1.050	0.997–1.106	0.067
	Hepatic flexure	1.052	0.998–1.110	0.059
	Large intestine, NOS	1.289	1.223–1.357	<0.001
	Rectosigmoid junction	1.143	1.088–1.201	<0.001
	Rectum	0.906	0.863–0.950	<0.001
	Sigmoid colon	0.996	0.949–1.045	0.858
	Splenic flexure	1.238	1.172–1.307	<0.001
	Transverse colon	1.087	1.034–1.143	0.001

### Stage-adjusted Cox model in cases with known stage

In the sensitivity analysis restricted to 195 324 tumors with known combined summary stage (localized, regional, distant), stage at diagnosis was strongly associated with CRC-specific mortality. Using localized disease as the reference, regional tumors had an approximately threefold higher hazard of CRC-specific death (HR = 3.17, 95% CI 3.08–3.26; *P* < 0.001), and distant tumors had about a 14-fold higher hazard (HR = 14.05, 95% CI 13.66–14.46; *P* < 0.001), after adjustment for age, sex, race, primary site, and surgery status. Female patients had a lower risk of CRC-specific death compared with males (HR = 0.93, 95% CI 0.92–0.95; *P* < 0.001). Relative to Black patients, those classified as White and Other (American Indian/Alaska Native or Asian/Pacific Islander) had lower hazards of CRC-specific death (HR = 0.87, 95% CI 0.84–0.89 and HR = 0.83, 95% CI 0.80–0.86, respectively; both *P* < 0.001), and the unknown race category also showed a reduced hazard (HR = 0.38, 95% CI 0.30–0.47; *P* < 0.001). Surgery status was also associated with CRC-specific mortality. Compared with patients who underwent cancer-directed surgery, those for whom surgery was not recommended, medically contraindicated, or refused/unknown had higher hazards of CRC-specific death (HR = 2.69, 95% CI 2.63–2.76; HR = 3.68, 95% CI 3.49–3.88; and HR = 2.88, 95% CI 2.76–3.01, respectively; all *P* < 0.001), and patients who died pre-operatively had the highest hazard (HR = 10.84, 95% CI 9.39–12.51; *P* < 0.001). Using appendiceal primaries as the reference site, all other major colonic and rectal subsites were associated with higher CRC-specific mortality (HRs generally 1.4–1.8; all *P* < 0.001) (Supplemental Digital Content Table S4, available at: http://links.lww.com/MS9/B226).

### Cause-specific Cox model in patients <50 years

In the sensitivity analysis restricted to patients younger than 50 years, several demographic and tumor-related factors remained significant predictors of CRC-specific mortality. Using White patients as the reference, Black patients had a modestly higher hazard of CRC-specific death (HR = 1.030, 95% CI 1.012–1.049; *P* = 0.001), whereas individuals categorized as Other (American Indian/Alaska Native or Asian/Pacific Islander) had a lower hazard (HR = 0.863, 95% CI 0.848–0.877; *P* < 0.001), and the small group with unknown race showed a markedly reduced hazard (HR = 0.214, 95% CI 0.182–0.251; *P* < .001). Sex had a minimal effect, with males exhibiting a slightly lower hazard than females (HR = 0.985, 95% CI 0.976–0.995; *P* = 0.002). Compared with adenocarcinoma, mucinous adenocarcinoma and signet-ring cell carcinoma were associated with higher CRC-specific mortality (HR = 1.272 and HR = 2.088, respectively; both *P* <0.001), while neuroendocrine/carcinoid tumors demonstrated substantially lower risk (HR = 0.188, 95% CI 0.178–0.198; *P* < 0.001), and other/rare histologies showed increased risk (HR = 1.258, 95% CI 1.233–1.284; *P* < 0.001). Relative to patients who received cancer-directed surgery, mortality hazards were significantly higher among those for whom surgery was not recommended (HR = 5.613, 95% CI 5.530–5.697), medically contraindicated (HR = 6.801, 95% CI 6.553–7.058), or not performed because the patient died pre-operatively (HR = 13.445, 95% CI 11.816–15.300), with elevated risk also observed in the refusal/unknown category (HR = 4.823, 95% CI 4.742–4.905; all *P* < 0.001). Using the appendix as the reference, most colonic subsites demonstrated higher hazards of CRC-specific death, including the ascending colon (HR = 1.084), cecum (HR = 1.328), descending colon (HR = 1.145), hepatic flexure (HR = 1.196), large intestine NOS (HR = 1.419), rectosigmoid junction (HR = 1.225), sigmoid colon (HR = 1.078), splenic flexure (HR = 1.368), and transverse colon (HR = 1.228), whereas rectal primaries did not differ significantly (HR = 0.963, 95% CI 0.918–1.010; *P* = .118). Together, these findings demonstrate that key prognostic patterns observed in the full cohort persist in younger patients (Supplemental Digital Content Table S5, available at: http://links.lww.com/MS9/B226).

## Discussion

Our comprehensive 47-year analysis of SEER data provides critical insights into long-term trends in CRC epidemiology and outcomes. Although survival improvements over time, a left-sided advantage, and poorer outcomes for signet-ring histology are consistent with prior reports, this 47-year SEER analysis provides long-term benchmarks from a fixed set of registries (1975–2022) and enables direct within-dataset comparisons across screening and treatment eras. We also report CRC-specific mortality using cause-specific Cox models, supported by modern-era stage-adjusted and early-onset (<50 years) sensitivity analyses. We observed that CRC overwhelmingly remains a disease of older adults, with more than half of patients diagnosed between the ages of 60–79 and fewer than 3% under the age of 40. This age distribution is consistent with global patterns, where CRC incidence rises sharply after midlife^[^13^]^. The sex distribution was nearly equal, although males had a slightly higher age-adjusted incidence (55.6 vs 42.5 per 100 000). Racial differences were evident: Black patients had the highest incidence rate in our cohort (51.7 per 100 000), while Asian/Pacific Islander and American Indian/Alaska Native patients had lower rates around 40 per 100 000, consistent with prior U.S. data^[^14^]^. Importantly, we found that colon cancers comprised approximately 79% of cases and rectal cancers 21%.

OS has improved markedly over the decades. OS improved across eras. Median OS was 3.83 years in 1975–1994, 5.58 years in 1995–2009, and 6.92 years in 2010–2022. Interpretation of the most recent era is made cautiously because follow-up is shorter and censoring is heavier. These gains in survival may reflect, in part, earlier detection and advances in treatment; however, causal attribution cannot be established using SEER registry data. We also found differences by anatomic site. Our cohort indicates that rectal cancer patients had longer median survival than colon cancer patients. This may be attributable to advances like total mesorectal excision and more effective multidisciplinary management of rectal tumors^[^15^]^. Notably, left-sided colon cancers were associated with significantly better survival than right-sided colon cancers in every era we examined. For example, in 1995–2009, we observed a median OS advantage of about 1.7 years for left-colon vs right-colon, and this gap persisted in 2010–2022, with left median survival of approximately 7.8 years vs right 5.7 years.

Histologic subtype was another key determinant of outcome. As expected, patients with conventional adenocarcinoma had intermediate prognoses, whereas those with mucinous adenocarcinoma fared slightly worse, and those with signet-ring cell carcinoma had the poorest survival. In our cohort, median OS for signet-ring carcinoma was only 1–2 years in each era, underscoring the aggressive nature of this subtype. This is in line with prior studies identifying signet-ring histology as an independent adverse prognostic factor^[^16^]^. In contrast, well-differentiated neuroendocrine tumors/carcinoids, which constituted a small percentage of cases, showed strikingly favorable outcomes. Many patients had prolonged survival beyond 10–20 years, reflecting the indolent behavior of low-grade NETs. Moreover, our analysis of treatment data confirms the life-prolonging benefit of surgery. Patients who underwent cancer-directed surgery had far superior survival than those who did not, whereas those for whom surgery was not performed due to advanced disease, contraindications, or refusal had very poor outcomes. This highlights that surgical resection of the primary tumor and metastases, when feasible, remains critical for long-term survival in CRC^[^17^]^.

Finally, in the full-cohort multivariable cause-specific Cox model for CRC-specific mortality (Table 2), female sex was associated with a modestly lower hazard of CRC-specific death compared with males (HR = 0.955, 95% CI 0.946–0.964; *P* < 0.001). Compared with White patients, Black patients had higher CRC-specific mortality (HR = 1.101, 95% CI 1.081–1.121; *P* < 0.001), whereas patients in the Other race category (American Indian/Alaska Native and Asian/Pacific Islander) had lower mortality (HR = 0.888, 95% CI 0.873–0.903; *P* < 0.001). The Unknown race category showed an implausibly low hazard (HR = 0.234, 95% CI 0.200–0.275; *P* < 0.001), which likely reflects missing-data or coding artifacts rather than a true biological association; therefore, we repeated the model excluding this category and found that results for the remaining race groups and other covariates were materially unchanged. Histologic subtype remained strongly associated with CRC-specific mortality: mucinous adenocarcinoma (HR = 1.277), signet-ring cell carcinoma (HR = 2.206), and other/rare histologies (HR = 1.221) were associated with higher mortality compared with conventional adenocarcinoma, whereas neuroendocrine/carcinoid tumors had substantially lower mortality (HR = 0.211; all *P* < 0.001). Surgery status showed the largest effect sizes, with markedly higher CRC-specific mortality when surgery was not recommended (HR = 5.631), contraindicated (HR = 6.229), not performed because of pre-operative death (HR = 12.822), or refused/unknown (HR = 4.609) compared with surgery performed (all *P* < 0.001). Age was independently associated with CRC-specific mortality, with a 1.3% increase in hazard per additional year of age (HR = 1.013, 95% CI 1.013–1.014; *P* < 0.001). Differences in outcomes by anatomic subsite have also been reported in prior CRC studies, with proximal tumors often demonstrating worse outcomes than distal tumors, supporting the biological and clinical heterogeneity of CRC by tumor location^[^18^]^.

### Comparison with previous literature

Our findings both corroborate and extend prior observations in the CRC literature. The overall incidence patterns we identified, with CRC primarily affecting older adults but with a concerning uptick in younger generations, are well documented in epidemiologic studies. Globally, CRC is the third most common malignancy and remains a leading cause of cancer death, with almost two million new cases and nearly one million deaths in 2020^[^13^]^. In the United States, CRC incidence and mortality have been on the decline in the over-50 population for several decades because of widespread screening and improved treatments^[^14^]^. Our data reflect this decline in older adults. However, consistent with recent reports, we observed an increase in incidence among adults under 50 as well as in the 50–54 age group. These age-specific trends underscore the public health importance of the 2021 U.S. Preventive Services Task Force recommendation to initiate average-risk CRC screening at age 45. At the same time, the continued rise in incidence before age 50 suggests that expanded screening alone may be insufficient, highlighting the need for prompt diagnostic evaluation of symptoms in younger adults and risk-stratified screening and surveillance strategies for individuals at higher baseline risk^[^19^]^. In our cohort, incidence among individuals <50 increased from 5.3 per 100 000 (1975–1994) to 8.2 (2010–2022), whereas incidence among individuals ≥50 declined from 206.6 to 110.9 per 100 000 over the same eras. This trend of early-onset CRC has been highlighted in numerous studies. Siegel *et al*, for example, noted approximately a 2% annual rise in CRC incidence in young adults in the United States in recent decades^[^14^]^. Particularly, the increase is disproportionate for rectal cancer: younger cohorts have experienced a high increase in rectal cancer rates, with annual percent increases of 3.2% in the 20–39 age group compared to colon cancer^[^20,21^]^. Our results echo this pattern, as we found a growing share of rectal tumors in the younger subset of patients over time.

Regarding tumor location, our study confirms previously observed differences between colon and rectal cancer outcomes. Population-based analyses have shown that when matched by stage and other factors, rectal cancer survival is often on par with colon cancer, and in some advanced-stage comparisons, rectal cancer even shows a slight advantage^[^22^]^. Lee *et al* analyzed SEER data (1995–2008). In stage IIB, colon cancer patients fared better than those with rectal cancer by a margin of four months. Patients with rectal cancer fared better than those with colon cancer in stages IIIC and IV by almost three months. Patients with stage IIB CRC had a worse prognosis than those with stage IIIA and IIIB disease. Colon cancer patients fared better than those with stage IIB rectal cancer after controlling for age, sex, and race; however, patients with stage IIIC and IV rectal cancer fared better than those with colon cancer^[^22^]^.

Within colon cancers, the prognostic divergence between left-sided and right-sided primaries is a well-established phenomenon that our study reinforces. Numerous studies, including a large meta-analysis by Petrelli *et al*, have demonstrated that left-sided colon cancers (distal to the splenic flexure) have significantly better survival than right-sided (proximal) colon cancers across stages. Petrelli’s analysis of 66 studies involving more than 1.4 million patients found an 18% reduced risk of death for left-sided tumors, independent of stage and treatment factors^[^23^]^.

Our analysis of histopathological subtypes also agrees with published literature. We found that mucinous adenocarcinomas had worse outcomes than non-mucinous adenocarcinomas, and signet-ring cell carcinomas had the worst outcomes of all. Prior research consistently shows that signet-ring cell carcinoma of the colorectal region is associated with a poor prognosis, often due to presentation at later stages and resistance to standard therapies^[^16^]^. For instance, a study by Yang *et al* reported a 5-year survival rate for signet-ring CRC of around 20–30%, which is significantly lower than that of typical adenocarcinomas^[^24^]^. Our observed median OS of only approximately 1 year in the 1975–1994 era and less than 2 years in 2010–2022 for signet-ring CRC is in line with its aggressive nature. In contrast, patients with well-differentiated neuroendocrine tumors had far better survival, reflecting the often-indolent course of localized carcinoid tumors. It is well known that appendiceal and rectal carcinoids can have 5-year survival rates well above 80–90% when localized, which matches the extended survival we observed for that group^[^25^]^.

Our results also resonate with historical trends in therapy development. The improvement in median and long-term survival we documented, particularly from Era 1 to Era 2, corresponds to the advent of effective adjuvant chemotherapy in the 1990s and improvements in surgical techniques. By the mid-2000s, further gains coincided with the introduction of new systemic agents (oxaliplatin, irinotecan) and targeted biologics (e.g., bevacizumab, cetuximab) for metastatic CRC^[^26,27^]^. Notably, immunotherapy with checkpoint inhibitors has produced remarkable outcomes in mismatch-repair-deficient (MSI-H) metastatic CRC, a fact reflected in contemporary trials. The pivotal KEYNOTE-177 study demonstrated that first-line pembrolizumab doubled median progression-free survival compared to chemotherapy in MSI-H metastatic CRC. Because these benefits depend on molecular subtype, equitable access to timely MSI/MMR testing and to immunotherapy when indicated is important to avoid widening outcome disparities^[^9^]^. Subsequent updates with over 5 years of follow-up showed a median OS of nearly 77.5 months (6.5 years) with immunotherapy, more than twice that of 36.7 months with standard chemotherapy, along with durable responses in a significant fraction of patients^[^9^]^. Our study’s most recent era likely includes patients benefiting from these advances (e.g., the tail of the survival curve in Era 3 may be prolonged by long-term immunotherapy responders).

### Strengths and limitations

This study leverages one of the largest population-based cancer registries available (*n* = 501 094) with 47 years of continuous coverage, providing exceptional statistical power to quantify long-term trends in incidence, subsite distribution, histology-specific outcomes, and era-to-era changes in survival. While shorter-term SEER studies provide valuable clinical snapshots, they are inherently limited in capturing the historical trajectory of the disease across paradigm shifts in care. A major contribution of this extended 47-year timeframe is the ability to benchmark survival and quantify the persistence of laterality- and histology-related prognostic differences across distinct screening and treatment eras, rather than relying on a single contemporary window. By utilizing a stable geographic cohort over nearly half a century, this multi-era approach uniquely demonstrates that, despite substantial improvements in OS driven by surgical and systemic therapy advancements, the relative prognostic penalties of right-sided location and aggressive histologies have remained stubbornly fixed. This continuous temporal perspective is essential for contextualizing modern survival estimates against historical baselines. The large sample and multi-decade span allow precise age-adjusted rate estimation and stable survival estimates for common subgroups and permit robust stratified analyses (left vs right colon, colon vs rectum, histologic subtypes, and surgical categories) that would be infeasible in single-institution cohorts. Use of standardized SEER coding and established analytical methods (direct age standardization, Kaplan–Meier survival, and Cox proportional hazards models) enhances reproducibility and facilitates direct comparison with prior population-based studies and screening/therapy-era benchmarks. Finally, the cause-specific Cox regression focusing on CRC death (with other causes censored) provides complementary inference to all-cause survival and helps isolate disease-related effects on mortality.

Several limitations inherent to registry-based research warrant emphasis. First, follow-up for patients diagnosed in 2010–2022 is substantially shorter than for earlier eras, leading to heavy right-censoring and limiting the stability of long-term estimates in the most recent era. Therefore, Era-3 survival estimates reflect early trends rather than definitive long-term outcomes and should be interpreted with caution. Second, SEER lacks several potentially important confounders and treatment details (performance status, comorbidity indices, detailed systemic therapy regimens, line of therapy, and molecular markers such as RAS/BRAF/MSI status for most years), which limits causal interpretation of treatment associations and contributes to residual confounding in multivariable models. In addition, stage at diagnosis could not be incorporated into the main survival analyses across 1975–2022 because consistent staging variables are only available in SEER from 2004 onward. We therefore performed 2004 + sensitivity analyses using combined summary stage (localized, regional, distant) in cases with known stage, which showed that regional and distant disease remained associated with higher CRC-specific mortality and that differences by primary site persisted after stage adjustment. In addition, because the SEER race recode aggregates American Indian/Alaska Native and Asian/Pacific Islander patients into a single “Other” category, we were unable to examine these groups separately; this may mask heterogeneity in CRC epidemiology and outcomes. Third, changes in coding practices, registry coverage, diagnostic intensity, and staging definitions over decades may introduce secular artifacts; although we applied consistent coding and carefully grouped eras to mitigate this, residual misclassification is possible. Fourth, cause-of-death attribution in registries can be imperfect, which may bias cause-specific survival estimates; therefore, results for cancer-specific mortality should be interpreted alongside all-cause survival. In addition, because we used cause-specific Cox models rather than competing-risks approaches such as Fine–Gray, our CSS results reflect cause-specific hazards and may overestimate the cumulative incidence of CRC-specific death in this elderly cohort where competing non-cancer mortality is common. Finally, because SEER 8 covers approximately 8% of the U.S. population, findings may not fully generalize to the entire U.S. population, and residual geographic selection bias cannot be excluded; external validation using other national datasets or pooled registries would strengthen generalizability. Our results reinforce the need for equitable implementation of organized CRC screening, including initiation at age 45 for average-risk adults and timely diagnostic work-up, especially for groups with persistently poorer outcomes. Consistent with contemporary global oncology models, these efforts should be embedded within multidisciplinary care pathways that integrate surgery, medical and radiation oncology, pathology, radiology, and supportive services.

## Conclusion

In this comprehensive 47-year SEER analysis, CRC survival improved substantially from the 1970s–1990s to the 1995–2009 era. Estimates for the most recent era are constrained by limited follow-up and should be interpreted cautiously. Survival varied meaningfully by anatomic subsite (rectum vs colon; left vs right colon), histologic subtype, and receipt of cancer-directed surgery, consistent with evolving surgical standards and systemic therapies. These findings underscore the importance of continued population-based surveillance, equitable implementation of screening and multimodality care, and incorporation of molecular and treatment-level data into future registry efforts to refine prognostic models and guide policy. Ensuring equitable access to effective screening and coordinated multidisciplinary care will be essential to sustain and extend the observed improvements in CRC outcomes.

## Data Availability

The data analyzed in this study were obtained from the National Cancer Institute’s SEER Program. Access to SEER Research Data is available through the SEER Data Access page upon completion of the required data-use agreement: https://seer.cancer.gov/data/access.html.

## Research registration unique identifying number (UIN)

Open Science Framework (OSF), https://doi.org/10.17605/OSF.IO/EU6ZG.
